# Texture Coding in the Rat Whisker System: Slip-Stick Versus Differential Resonance 

**DOI:** 10.1371/journal.pbio.0060215

**Published:** 2008-08-26

**Authors:** Jason Wolfe, Dan N Hill, Sohrab Pahlavan, Patrick J Drew, David Kleinfeld, Daniel E Feldman

**Affiliations:** 1 Department of Physics, University of California San Diego, La Jolla, California, United States of America; 2 Section on Neurobiology, Division of Biological Sciences, University of California San Diego, La Jolla, California, United States of America; Harvard University, United States of America

## Abstract

Rats discriminate surface textures using their whiskers (vibrissae), but how whiskers extract texture information, and how this information is encoded by the brain, are not known. In the resonance model, whisker motion across different textures excites mechanical resonance in distinct subsets of whiskers, due to variation across whiskers in resonance frequency, which varies with whisker length. Texture information is therefore encoded by the spatial pattern of activated whiskers. In the competing kinetic signature model, different textures excite resonance equally across whiskers, and instead, texture is encoded by characteristic, nonuniform temporal patterns of whisker motion. We tested these models by measuring whisker motion in awake, behaving rats whisking in air and onto sandpaper surfaces. Resonant motion was prominent during whisking in air, with fundamental frequencies ranging from approximately 35 Hz for the long Delta whisker to approximately 110 Hz for the shorter D3 whisker. Resonant vibrations also occurred while whisking against textures, but the amplitude of resonance within single whiskers was independent of texture, contradicting the resonance model. Rather, whiskers resonated transiently during discrete, high-velocity, and high-acceleration slip-stick events, which occurred prominently during whisking on surfaces. The rate and magnitude of slip-stick events varied systematically with texture. These results suggest that texture is encoded not by differential resonant motion across whiskers, but by the magnitude and temporal pattern of slip-stick motion. These findings predict a temporal code for texture in neural spike trains.

## Author Summary

A fundamental problem in neuroscience is understanding how behaviorally relevant information is collected by a sensory organ and subsequently encoded by the brain. By actively moving their whiskers, rats can discriminate fine differences in textures. Little is known, however, about how whisker dynamics reflect texture properties or how the nervous system encodes this information. In one hypothesis, whisker motion over a texture produces a unique, texture-specific temporal profile of velocity, which is encoded in the temporal pattern of neural activity. In a second, alternative hypothesis, textures excite a specific subset of whiskers due to intrinsic, whisker-specific mechanical resonance frequencies. Information is then encoded by the spatial distribution of neural activity in whisker-related columns in cortex. Here, we assess these hypotheses by measuring whisker motion as animals whisk across sandpapers of varying roughness. We found that whiskers resonate in air and on surfaces, but that these resonance vibrations do not vary with, and therefore do not encode, texture. Instead, whisker motion over a textured surface produces fast, transient slip-stick events whose dynamics are dependent on texture roughness. Texture is likely to be encoded in the temporal pattern of spikes evoked by these slip-stick events.

## Abstract 3

How rats use their whiskers to sense surface texture has been unclear. Measurement of whisker motion in awake-behaving rats indicates that texture information is read out by the pattern of discrete, slip-stick whisker motion events, and not by differential whisker resonance, as had been hypothesized previously.

## Introduction

Rodent whiskers, like human fingertips, are tactile detectors that are actively moved through the environment to sense position, shape, and surface features of objects. A particularly salient surface feature is texture, which is more readily distinguishable using touch than vision [1]. Rats discriminate textures using their whiskers with a precision that rivals human fingertips [2–5]. How whiskers read out texture information, and how that information is encoded in the nervous system, are vigorously debated, and have important implications for sensory processing in the whisker system [6,7], which is a major model system for studying cortical function and plasticity [8,9].

Rats have an array of approximately 30 large whiskers (macrovibrissae) on each side of the face. Whisker length varies systematically across the whisker pad, with caudal whiskers being longer than rostral whiskers. Whiskers are moved rhythmically at 5–15 Hz to explore objects in the environment, including textures [5,6,10]. Two main hypotheses exist for texture discrimination by the whiskers, based on experiments using detached whiskers and in anesthetized animals. The resonance hypothesis derives from the observation that whiskers are resonant beams, with characteristic resonance frequency inversely related to whisker length [11,12]. Whisker-length variation across the whisker pad results in a spatial map of fundamental resonance frequency (FRF). In this hypothesis, whisker-tip motion across surface microfeatures causes tip vibration at a frequency that varies with texture spatial frequency. Only when textures generate tip vibration at the FRF will vibrations most effectively build up and be transmitted to the whisker follicle, where transduction occurs. As a result, each whisker is best activated by a specific range of textures, and each texture preferentially activates a subset of whiskers, leading to a spatial code for texture in the relative amplitude of vibrations across the whisker array [13]. An alternative model is that texture is encoded temporally, by unique temporal patterns of movement (“kinetic signatures”) that are induced within single whiskers scanning across surfaces. These patterns have been proposed to include both mean speed (amplitude × frequency) of whisker vibration [7,14], spectral composition of whisker vibrations [15], and the precise, irregular velocity profile of whisker motion [7]. This latter feature provides higher-resolution texture information than vibration speed or frequency alone [7,16].

To distinguish these models, it is critical to measure whisker vibrations and neural responses in awake, behaving animals voluntarily palpating surfaces. This is because the dynamics of voluntary whisker movement will critically impact the transformation of surface features into whisker-motion signatures. Whiskers are known to exhibit multiple modes of vibration during voluntary palpation of surfaces, including resonance vibration and irregular, high-velocity motion events [17]. However, which of these features correlate with, and therefore may encode, texture, is not known.

Here, we evaluated the resonance and kinetic signature models of texture by precisely measuring whisker vibrations in awake, behaving rats trained to actively whisk onto textured surfaces. Results showed that whisker resonance occurs during free whisking in air and during brief, discrete epochs while whisking onto textures. However, the magnitude of resonance vibrations did not vary across textures, as required for the resonance hypothesis. Instead, whisker resonance on surfaces primarily represented transient ringing during brief (5–10 ms), high-velocity, high-acceleration slip-stick events. Slip-stick events were a prominent component of whisker motion on surfaces, and the rate and magnitude of these events correlated well with texture. These results indicate that whisker resonance occurs in awake rats and shapes natural whisker vibrations, but that texture is not encoded by differential resonance across whiskers, at least under these behavioral conditions. Instead, slip-stick events may contribute to a kinetic signature for texture in the whisker system.

## Results

### Behavioral Training and Measurement of Whisker Movement

**Figure 1:**
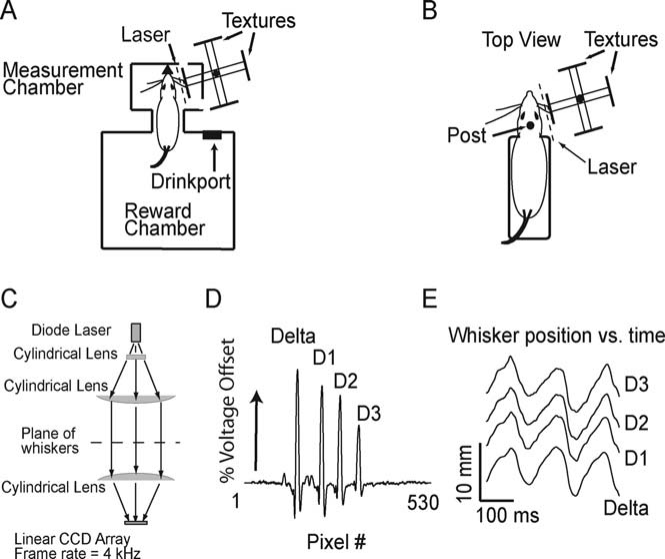
Training and Measurement Methods (A) Training environment for whisking while in the nose poke. Black triangle, nose poke. Textures were mounted on a four-arm holder on a stepper motor, and were rotated into place between trials. Whisking in air was measured by omitting a texture from one of the arms. A plane of laser light (generated by the lens system in [C]) was projected from above, down through a slit in the floor, and onto a linear CCD imaging array below the cage. (B) Training setup for whisking in head-fixed animals. Animals were accommodated to being held in a Plexiglas tube and head-fixed via a post fixed to the tube. The head was placed in the same position and orientation relative to the surface and CCD imaging array as for animals whisking in the nose poke. (C) Side view of optical system for generating and tracking whisker shadows. Light from a diode laser was collimated into a line (1-mm wide, 60-mm long), projected onto the whiskers from above, and focused onto the linear CCD array below the training cage (D) Example of whisker shadows (voltage peaks) in a single-frame output of the CCD array. (E) Whisker motion over time revealed by tracking voltage peaks of four whisker shadows simultaneously.

To measure whisker movement in awake, behaving rats, we trained rats to whisk in air and against textured surfaces. Two behavioral paradigms were used. In Behavior 1, six rats (N1–N6) positioned their nose in a small aperture (the nose poke) and whisked in air and onto surfaces for approximately 0.5 s to receive a water reward (Figure 1A). Textured surfaces (sandpapers of varying roughness, mounted on aluminum backing) were positioned statically in the whisking path of the right whiskers using a computer-controlled stepper motor. Whisker motion in the protraction–retraction plane (roughly rostrocaudal, parallel to the face) was measured optically from whisker shadows cast by a collimated plane of laser light onto a linear charge-coupled device (CCD) imaging array below the training cage (Figure 1C–1E). Each trial consisted of whisking either in air or onto one surface, and lasted 491 ± 179 ms. Between trials, rats moved to a separate chamber to receive a water reward, and surfaces were changed using the stepper motor. Rats performed 123 ± 43 (mean ± standard deviation [s.d.]) trials per daily session. In Behavior 2, four rats (H1–H4) were habituated to being transiently head-fixed, and whisked voluntarily in air and onto surfaces (Figure 1B). During each daily session (15–30 min), rats performed 69 ± 36 trials, with a trial defined as a 3-s epoch that included a variable duration of whisker motion. See Materials and Methods for training techniques.

In both behaviors, motion of one to four identified whiskers was tracked at 4-kHz frame rate and approximately 5-μm spatial resolution, using the linear CCD array. Because whisker shadows did not cross during whisking, up to four whiskers could be identified and tracked simultaneously using automated software (Figure 1D). Nonimaged whiskers were trimmed weekly at the base. Surfaces were presented parallel to the face, less than 5 mm from the whisker tips. Whisker motion was tracked 6–14-mm (typically 10 mm) from the face (for whisking in air), and halfway between the surface and the whisker pad (for whisking onto textures). All training and whisker measurements were performed under computer control using custom-written programs in Labview (National Instruments).

### Whiskers Vibrate at High Frequency during Voluntary Whisking in Air

**Figure 2:**
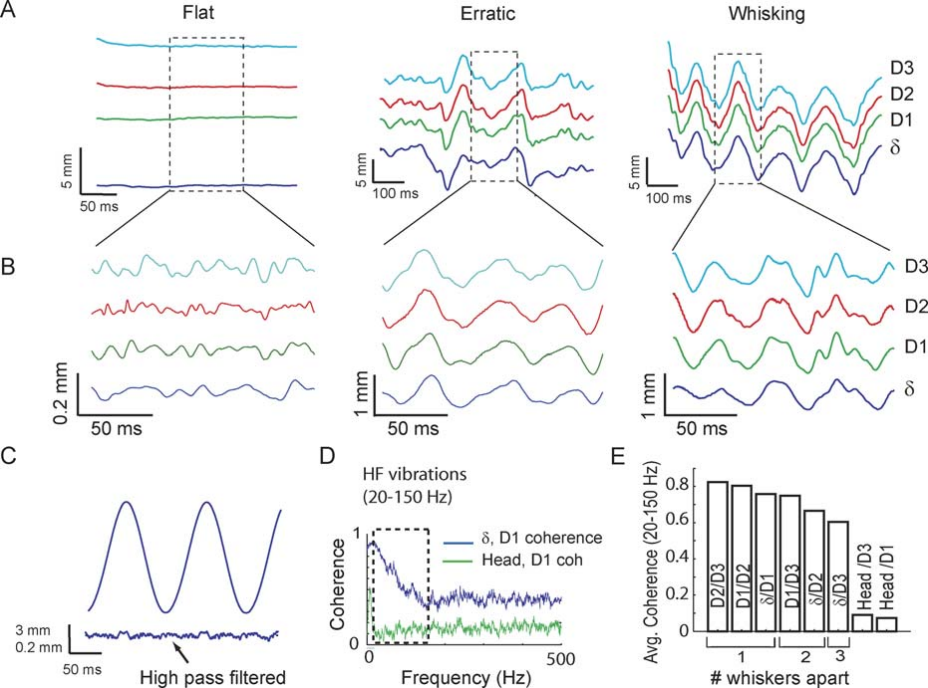
High-Frequency Vibrations Are Present during Natural Whisker Motion in Air. (A) Example epochs showing a stationary period (“flat”), erratic motion, and rhythmic whisker motion, for δ, D1, D2, and D3 whiskers measured simultaneously. (B) Bandpass-filtered (20–1,000 Hz) position traces for the segments shown in (A), showing high-frequency (HF) motion. (C) HF vibrations are absent in a mechanically driven whisker moving sinusoidally at 8 Hz. (D) Spectral coherence of D1 whisker motion and head motion in rat N4, and between D1 whisker and δ whisker motion. Dashed region indicates frequency range of HF vibrations (20–150 Hz). (E) Quantification of mean coherence in the 20–150 Hz frequency band between different whiskers, and between whisker motion and head motion, for rat N4.

We first tested the resonance hypothesis by asking whether whiskers resonate, and whether a map of resonance frequency exists, in awake, behaving rats whisking in air. For rats whisking in the nose poke (Behavior 1), whisker motion typically included periods of regular, 5–15 Hz whisking, periods when the whiskers were held stationary, and periods of erratic motion. Examples are shown in Figure 2A. During all three types of motion, bandpass filtering (20–1,000 Hz) revealed prominent high-frequency whisker vibrations (20–150 Hz), with approximate peak-to-peak amplitude of 0.1 to 0.5 mm, that were superimposed on the low-frequency motion (Figure 2B).

These high-frequency vibrations were not apparent during motion of an isolated whisker attached to an electric motor moving sinusoidally at 8 Hz (Figure 2C), indicating that they were not due to external vibrations in the recording apparatus or to interaction between the moving whisker and air. To test whether vibrations were due to head motion, versus whisker motion relative to the head, we simultaneously measured head and whisker motion in one rat (N4) by attaching a horizontal bar to the top of the skull. The bar cast a shadow on the CCD array that could be tracked independently of the whisker shadows. Head motion and D1 whisker motion showed coherence at low frequencies (<8 Hz), but very little coherence (mean 13%) at frequencies greater than 10 Hz (Figure 2D, green trace). The same was true for head motion and D3 whisker motion (Figure 2E). Thus, head motion is not the source of high-frequency whisker vibration at greater than 20 Hz. Consistent with this conclusion, high-frequency whisker vibrations were also prominent in head-fixed rats whisking in air (unpublished data).

Despite the lack of coherence between whisker motion and head motion, neighboring whiskers exhibited high coherence in the 20–150 Hz range (Figure 2D and 2E). This suggests a common driving force for high-frequency vibrations across whiskers. Coherence between whiskers decreased slightly with whisker separation on the face, suggesting that neighboring whiskers receive the greatest common drive (Figure 2E). Together, these measurements indicate that high-frequency, coherent vibrations occur during free whisking in air, superimposed on low-frequency whisking motion.

### Whisker Resonance Is Evident from High-Frequency Vibrations

**Figure 3:**
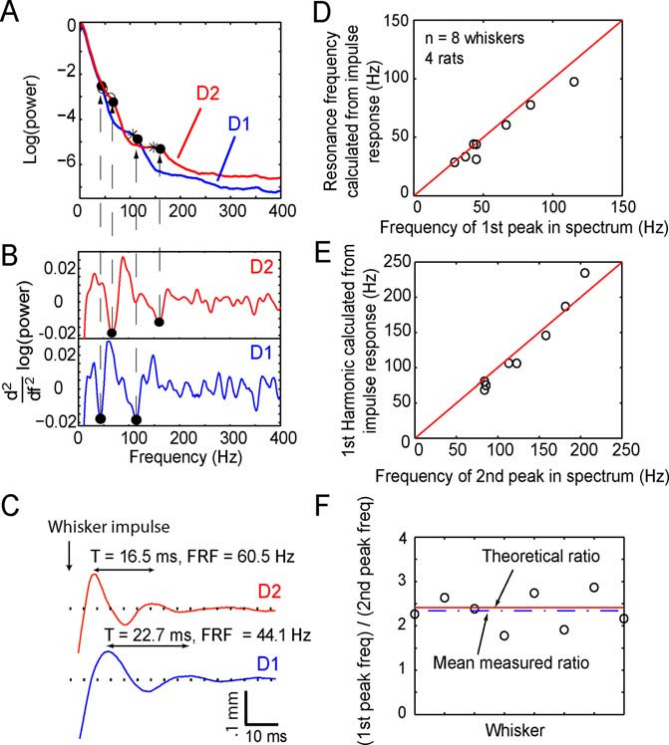
Whiskers Preferentially Vibrate at Resonance Frequency during Whisking in Air (A) Power spectra of whisker motion during whisking in air for the D1 and D2 whiskers of rat N2. Filled circles denote shoulders, identified as the points of maximum negative concavity (minima in the second derivative of the power spectra). Open circles and asterisks show the FRF and calculated first harmonic (respectively), measured by the impulse method in the anesthetized animal, after the behavioral recording session. (B) Second derivative of the power spectra in (A). (C) Impulse method for measuring FRF in the anesthetized animal, for the whiskers in (A). FRF is calculated from the period (T) of ringing after delivering a sharp impulse. (D) Relationship between FRF calculated by the impulse method and first peak of the power spectrum during whisking in air, for eight whiskers in four rats performing the nose poke task. (E) Relationship between calculated first harmonic of the FRF, measured by impulse method, and the second peak of the power spectrum during whisking in air. (F) Measured ratio of first peak to second peak in the power spectrum during whisking in air, for eight whiskers in four rats performing the nose poke task. Dashed line indicates the mean ratio. Solid line indicates the theoretical value of 2.41 for *f*_1_/FRF ratio for a conical beam.

We tested for whisker resonance during free whisking in air by examining the relationship between the frequency spectrum of whisker vibrations in air and the intrinsic resonance frequencies of the whiskers. Experiments were performed in rats N1–N4 performing the nose poke task (Behavior 1). Power spectra during whisking in air were calculated for each whisker across 44–122 trials. Power spectra were not smooth, but rather showed modest peaks and shoulders representing dominant frequencies of whisker vibration. Two such power spectra, from the D1 and D2 whiskers imaged simultaneously in rat N2, are shown in Figure 3A. Peaks and shoulders were identified precisely as minima in the second derivative of the logarithm of the power spectra, which correspond to points of negative concavity (filled circles in Figure 3A and 3B).

After whisking in air, rats were anesthetized; the intrinsic resonance frequency for each whisker was directly measured by manually delivering an impulse to the whisker, and the FRF from the resulting decaying oscillations in air was calculated, as imaged on the CCD array (see Materials and Methods). Examples are shown in Figure 3C for the D1 whisker (length: 38.7 mm, FRF: 44.1 Hz) and D2 whisker (length: 31.6 mm, FRF: 60.5 Hz) from rat N2 (same whiskers as for the power spectra in Figure 3A). Theoretical first harmonics of the FRF were calculated as (10.6/4.4) × FRF, as predicted for a conical beam model of the whisker (see Materials and Methods). For this rat, peaks in the power spectra during voluntary whisking in air (filled circles) were found to align well with the measured FRFs (open circles) and calculated first harmonics (asterisks) obtained by the impulse method in the anesthetized animal (Figure 3A).

Across eight whiskers in rats N1–N4 (*n* = 4 rats), the first peak in the power spectra during natural, active whisking aligned well with the measured FRFs (Figure 3D), and the second peak aligned with the predicted first harmonics (Figure 3E). Moreover, the ratio of the frequencies of the first and second peaks in the power spectra was 2.35 ± 0.14 (*n* = 8), close to the theoretical ratio of 10.6/4.4 = 2.41 for *f*_1_/FRF for a conical beam. Thus, during natural whisking in air, whiskers preferentially vibrated at the FRF and its first harmonic, though the magnitude of these vibrations was small.

**Figure 4:**
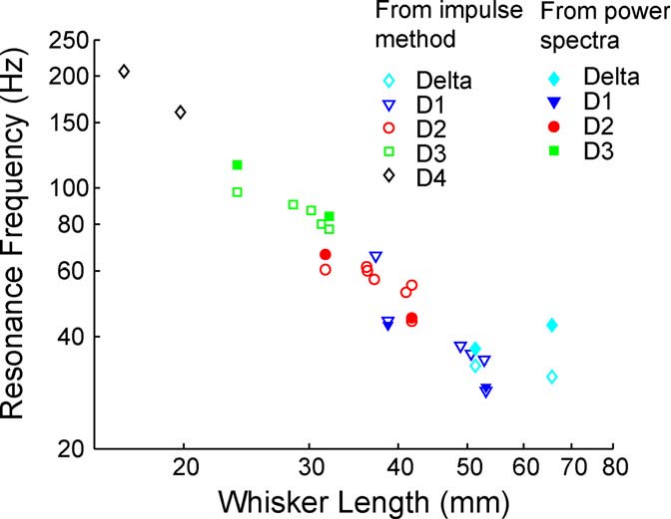
Map of Resonance Frequency under Anesthesia and during Whisking in Air Resonance frequency versus whisker length for all whiskers measured, plotted on a log-log scale. Filled symbols, fundamental resonance frequency (FRF) during whisking in air, calculated as the first peak in the power spectrum during active whisking (same data as in Figure 3). Open symbols, FRF measured by the impulse method in anesthetized rats (includes data from Figure 3 and from 13 additional whiskers).

To test whether a map of whisker resonance exists across the whisker pad in the awake, behaving rat, we compared first peaks in the power spectra during whisking in air and FRFs measured by the impulse method, to whisker length (Figure 4). We found a systematic relationship in which longer whiskers exhibited lower FRFs and vibrated preferentially at these lower frequencies during whisking in air. This indicates that a map of resonance frequency exists across the whiskers in awake, whisking animals, as predicted by the resonance hypothesis [11–13].

### Resonance Filtering of Whisker Vibrations Confirmed by Whisker Trimming

**Figure 5:**
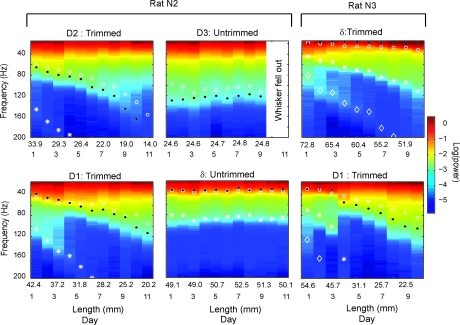
Whisker Trimming Causes Power Spectra during Whisking in Air to Shift Systematically to Higher Frequencies Power spectra for whisker motion in air (colored vertical bars) measured daily in two animals during progressive whisker trimming. Rat N2 had the D1 and D2 whiskers trimmed approximately 2 mm per day. Rat N3 had the δ and D1 whiskers trimmed 2–4 mm per day. The D3 and δ whiskers of rat N2 remained untrimmed. Open circles, whisker FRF measured daily by the impulse method. Asterisks and diamonds, first and second harmonics of the measured FRF calculated using the truncated cone model of the whisker. Black dots are the calculated first shoulders of the power spectra (see Figure 3). Whisker length was measured daily, but for clarity, only alternate days' measurements are shown in the figure.

To confirm the role of whisker resonance in shaping high-frequency vibrations in air, we systematically altered whisker resonance by trimming whiskers in two rats (N2 and N3). Power spectra were measured daily, for 10–11 d, during whisking in air for whiskers δ, D1, D2, and D3. In rat N2, the D2 and D1 whiskers were trimmed by approximately 2 mm after each day's measurement, while δ and D3 whiskers were left untrimmed. In rat N3, δ and D1 were trimmed 2–4 mm shorter each day. Whisker length was measured daily. Results are shown in Figure 5. The power spectrum for whisking in air on each day is presented as a color plot in each vertical strip. Whisker FRF was measured daily using the impulse method, and first and second harmonics of the FRF were calculated using a model of the whisker as a truncated (i.e., trimmed) conical beam, rather than an intact conical beam ([18]; see Materials and Methods). The FRF and first and second harmonics, calculated from the impulse measurements, are plotted as open circles, asterisks, and diamonds, respectively, on each day's power spectra.

Results showed that as trimming decreased whisker length, power spectra for whisking in air shifted systematically towards higher frequencies, as expected if resonance filtering shaped whisker vibrations. Trimming shifted the shoulders of the power spectrum (black filled circles) in parallel with the resonance frequencies (FRF and harmonics) measured by the impulse method. This was particularly evident for the D1 whisker in rat N2 and the δ whisker in rat N3, where bands of amplification (shoulders) in the power spectra closely followed the resonance frequencies measured by the impulse method. In contrast, power spectra remained stable for untrimmed whiskers, measured simultaneously in the same behavioral trials.

In a converse experiment (*n* = 1 rat), the D2 whisker was trimmed substantially, and then allowed to regrow by 12 mm over 14 d. Power spectra for whisking in air were measured before regrowth (when the whisker was trimmed) and afterwards. Results showed that regrowth was accompanied by a pronounced shift in the power spectrum of the D2 whisker towards lower frequencies, without substantial changes in the power spectra for nearby, simultaneously measured, untrimmed whiskers, whose length did not change appreciably during the regrowth period (unpublished data).

Together, these results indicate that resonance properties of whiskers shape high-frequency (>20 Hz) whisker vibrations during natural free whisking in air. This suggests that whisker resonance may be a relevant mechanism for filtering whisker input during active whisking in awake animals.

### Source of High-Frequency Whisker Vibrations in Air

High-frequency whisker vibration in air is not due to head movement (Figure 1D and 1E), and therefore is likely to reflect high-frequency drive by whisker muscles. High-frequency muscular drive is plausible because high-frequency (83 Hz) electrical stimulation of motor axons in the facial nerve can cause whisker movements at stimulation frequency [19]. We observed high-frequency whisker vibrations in response to facial nerve stimulation in anesthetized rats, and found that evoked vibrations can strongly drive whisker resonance (Figure S1).

To test whether whisker muscles drive high-frequency whisker vibrations in awake, whisking rats, we measured electromyogram (EMG) activity from whisker muscles while imaging whisker motion in air (*n* = 5 rats). EMG was measured from intrinsic muscles and the extrinsic muscle *m. nasolabialis*, which drive whisker protraction and retraction, respectively, during whisking [20,21]. In different rats, EMG was measured from *m. nasolabialis*, intrinsic muscles, or both simultaneously, together with the movement of one or two different whiskers (Table 1). In total, four *m. nasolabialis* EMG recordings were obtained simultaneously with movement of seven whiskers, and three intrinsic EMG recordings were made simultaneously with movement of six whiskers.

**Table 1:**
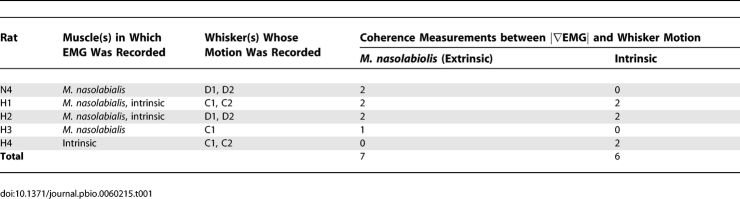
EMG Measurements

**Figure 6:**
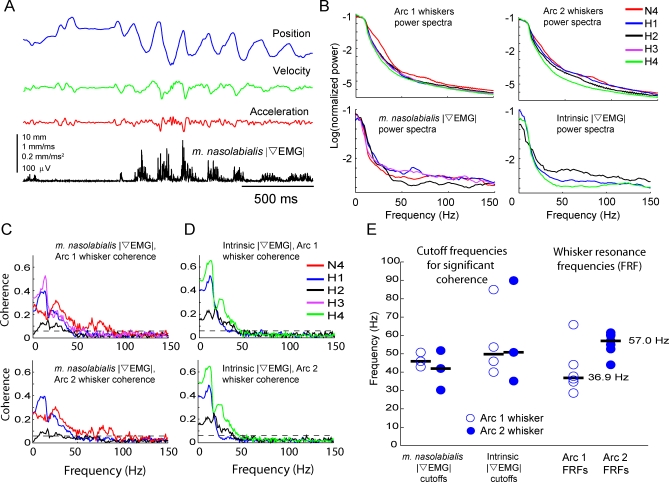
Coherence between Whisker Vibration and |∇EMG| of Whisker Muscles during Whisking in Air (A) Example |∇EMG| from an extrinsic muscle and movement of the D1 whisker (rat H3). (B) Power spectra for whisker motion and |∇EMG| for all whiskers and muscles measured. Legend indicates animal identity. Each power spectrum was normalized to its total power. (C) Coherence between *m. nasolabialis* |∇EMG| and arc 1 whiskers (top) and arc 2 whiskers (bottom). Each line is coherence between one |∇EMG| recording and one whisker, recorded in one animal. Dashed black line shows *p* = 0.05 significance level. Coherence traces are color coded according to animal. (D) Coherence between intrinsic |∇EMG| and whisker motion. Plotted as in (B). (E) Cutoff frequencies for significant coherence between |∇EMG| recordings and whisker motion (frequency at which coherence dropped below significance), compared to measured FRFs for the same whiskers.

EMG activity was coherent with whisking (Figure 6A), as previously reported [20,21], with intrinsic muscles generally active during protraction and *m. nasolabialis* active during retraction (unpublished data). The rectified, differential EMG (|∇EMG|) power spectra revealed high-frequency muscle activity up to 50 Hz (Figure 6B). To determine whether high-frequency muscle activity drove high-frequency whisker movement, we measured the spectral coherence between |∇EMG| and whisker position in air, during all types of whisker motion (whisking, erratic, and flat). For both intrinsic and extrinsic muscles, coherence between |∇EMG| and whisker motion was generally statistically significant, with values between 0.15 and 0.65, for frequencies less than 50 Hz, and fell below significance by approximately 50 Hz (Figure 6C and 6D). This was true for both arc 1 (D1 and C1) whiskers, and arc 2 (D2 and C2) whiskers. The maximal frequency of significant coherence, termed the cutoff frequency, was defined as the frequency at which coherence magnitude fell below the *p* = 0.05 significance level for nonzero coherence (see Materials and Methods for confidence interval calculation). For arc 1 whiskers, cutoff frequency was less than approximately 50 Hz for six of seven measurements, and approximately 90 Hz in the remaining measurement (Figure 6E). Thus, muscle activity was significantly, but only modestly, coherent with whisker motion at the FRF of arc 1 whiskers (median measured FRF: 36.9 Hz). Coherence at the FRF was even weaker for arc 2 whiskers, which also showed cutoff frequency of less than approximately 50 Hz in six of seven cases, and had a median FRF of 57.0 Hz (Figure 6E). We conclude that whisker muscles provide some high-frequency energy that could drive whisker vibrations, but because coherence was weak at high frequencies, how muscle contractions drive high-frequency vibrations remains unresolved.

### Whiskers Resonate on Textured Surfaces, but Differential Resonance Does Not Encode Texture

The above results indicate that resonant motion is prominent during whisking in air, and that a map of resonance frequency exists across the whiskers. To determine whether this resonance map is used to encode surface texture, we explicitly tested the two central predictions of the resonance model for texture coding: first, that whiskers resonate at distinct, characteristic resonance frequencies as they sweep across surfaces; and second, that the amplitude of resonance frequency vibrations in each whisker depends on surface texture, resulting in one preferred texture that drives the strongest vibrations. Together, these properties have been proposed to result in a spatial map of texture across the whiskers [11,22].

We measured whisker motion on sandpaper surfaces in three rats performing the nose poke task (rats N4–N6) and two rats that whisked while head-fixed (rats H1–H2). We used seven sandpapers: P150 (roughest), P240, P400, P600, P800, P1200, and P1500 (finest). These correspond to 100-, 58-, 35-, 26-, 22-, 15-, and 13-μm mean particle size. Rats can readily distinguish two coarse sandpapers [3], a smooth surface from P100 sandpaper [10], and can distinguish 60-μm differences in spacing of periodic grooves [5], suggesting that differences between these sandpapers (or at least between the roughest and smoothest sandpapers) should be discriminable using the whiskers. Up to four sandpapers were presented per measurement session, typically in blocks of five to ten trials each. Different subsets of sandpapers were presented on different days. Surfaces were placed parallel to the whisker pad, 5 mm closer to the face than the whisker length. (Because whisker tips move in an arc, this meant that approximately 5 mm of whisker tip contacted the surface at mid-whisk, and less than 5 mm contacted at maximum protraction and retraction). Because whiskers are different lengths, we measured movement of only a single whisker at a time across the surfaces. We verified continuous whisker–surface contact during whisking in each animal, by observing the presence of a consistent whisker shadow on the CCD imaging array when the array was positioned 1 mm from the surface. It was not possible to position surfaces closer to the whisker tip, given the lateral freedom of head position within the nose poke (approximately 2 mm). Whisker motion was measured halfway between the surface and the whisker pad (∼10 mm from the follicle).

**Figure 7:**
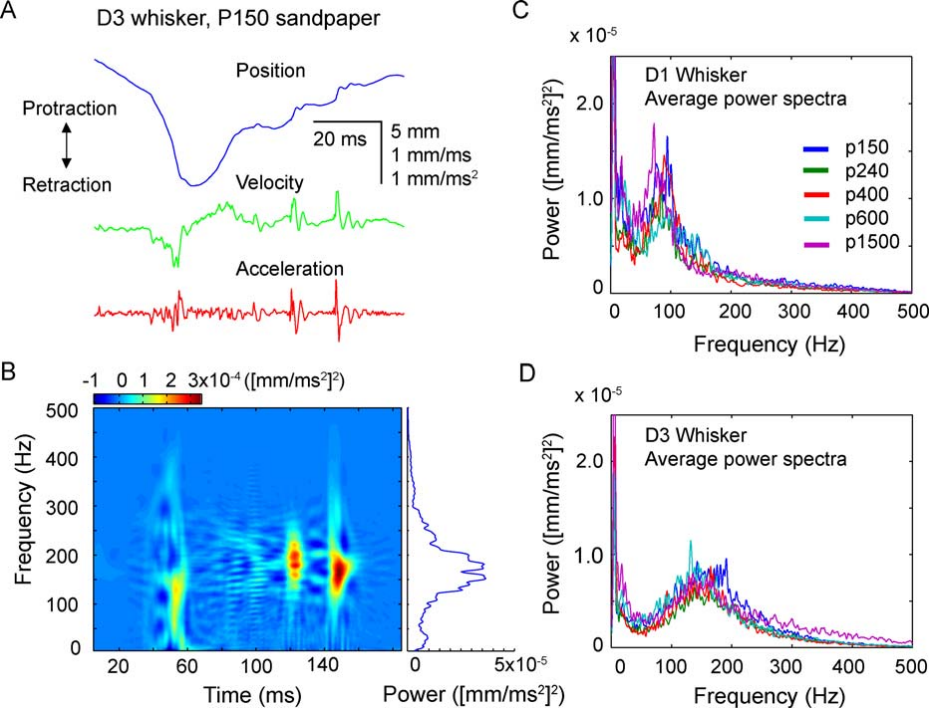
Whisker Resonance during Movement on Textured Surfaces (A) D3 whisker position, velocity, and acceleration during one retraction–protraction cycle on P150 sandpaper (rat N1). (B) Time-frequency representation (TFR; color plot) of the whisker acceleration shown in (A). The trace on the right is the integrated TFR across the trial, which is equal to the average power spectrum. (C) Average power spectra (integrated TFRs) for D1 whisker motion for all protraction and retraction epochs onto five textures for rat N1, showing broad, high-frequency peak at approximately 80 Hz. (D) Average power spectra for D3 whisker motion onto the same five textures for rat N1, showing high-frequency peak at approximately 150 Hz.

An example of whisker motion across a rough (P150) sandpaper is shown in Figure 7A. The rat initially retracted the D3 whisker across the surface (negative slope in the position trace), and then protracted it (positive slope). Whisker velocity and acceleration, calculated from the position trace, revealed approximately three brief, high-acceleration, high-velocity events that occurred during whisker motion. To analyze the time-varying spectral content of whisking on the surface, we calculated the Wigner-Ville time-frequency representation (TFR), qualitatively similar to a spectrogram, for this whisker motion (Figure 7B). The TFR showed prominent, brief epochs of vibration at approximately 150–180 Hz, aligned with the rapid movement events. The integrated TFR across the entire whisking period (which is equal to the average power spectrum) revealed a broad peak at approximately 150–180 Hz (Figure 7B, rightmost trace). Similar broad peaks at 50–180 Hz were observed for D1, D2, and D3 whiskers moving across a variety of surfaces (see below).

We tested whether these broad, high-frequency peaks were consistent with whisker resonance by comparing the peak frequencies across different length whiskers. (The FRF while the whisker is pinned against a texture will not equal the FRF measured in air, because boundary conditions for vibration are changed and the whisker is effectively shortened [11,23,24]). Figure 7C and 7D show power spectra for vibrations of the D1 and D3 whiskers of rat N4, measured during palpation on five different sandpapers. Power spectra were calculated as integrated TFRs for all individual protraction and retraction epochs on a given surface, and then averaged across these epochs to obtain the average power spectrum for each surface. Consistent with the resonance model, the D1 whisker showed a high-frequency vibration peak at approximately 80 Hz, while the shorter D3 whisker showed a peak of approximately 150 Hz (Figure 7C and 7D). We repeated this analysis for ten whiskers (five D1 whiskers, four D2 whiskers, and one D3 whisker) in five rats (N4–N6, H1, and H2). We calculated the high-frequency peak of the average power spectrum for each whisker moving on each texture (identified as the first peak in the power spectrum >40 Hz).

**Figure 8:**
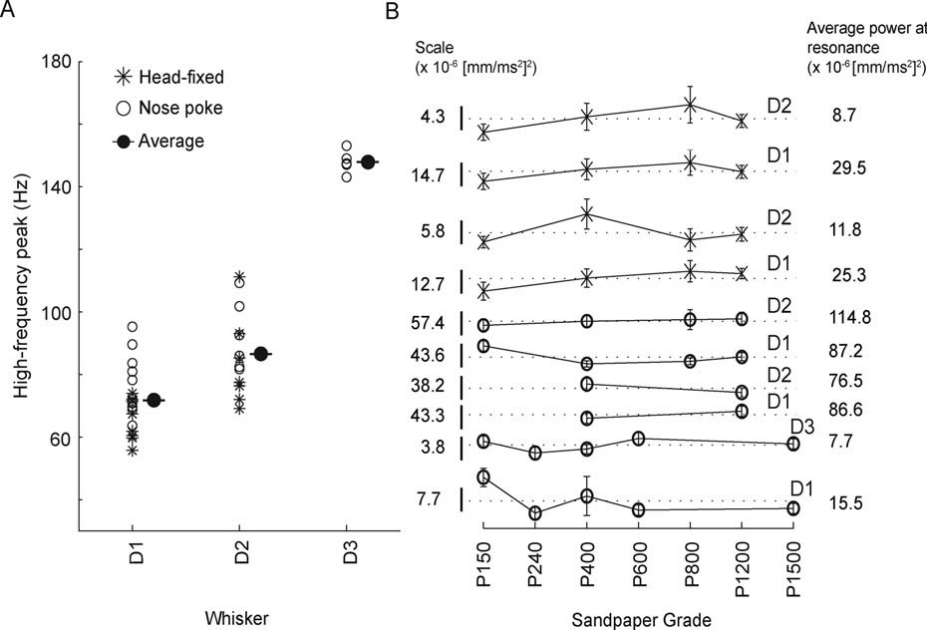
Population Data for Whisker Resonance on Different Textured Surfaces (A) High-frequency peak of the average power spectrum for all whiskers and all surfaces (asterisks indicate head-fixed animals, circles indicate nose poke animals). Each point is the average peak frequency of one whisker on one surface. Filled circles indicate the average across all individual measurements. (B) Average power at the presumed resonance frequency (high-frequency peak of the average power spectrum) as a function of texture, for each whisker for which measurements on multiple textures were made. Traces are offset vertically for clarity. Scale bars (left) show power for each trace. Dashed lines and right-hand numeric values show average power across textures (scale bar length is 50% of the average power). Average power did not substantially or systematically vary with texture.

Across all textures, the high-frequency peak for D1 whiskers was found to be between 57.7 and 91.2 Hz (mean: 71.8 Hz); for D2 whiskers, 68.5–114.0 Hz (mean: 86.6 Hz); for the single D3 whisker, 142.2–153.9 Hz (mean: 147.9 Hz) (Figure 8A). Thus, for both animals performing the nose poke behavior (open circles, Figure 8A and 8B) and head-fixed animals (asterisks, Figure 8A and 8B), measured peaks in vibration power spectra were at higher frequencies for the shorter whiskers and lower frequencies for the longer whiskers, consistent with intrinsic resonant properties of the whiskers. These data therefore suggest that whiskers vibrate at characteristic resonance frequencies when moving across surfaces, at least when distance to the surface is kept constant. Subsequent analyses assume that the high-frequency vibration peak represented the whisker's resonance frequency on surfaces. Power at the high-frequency peak during whisking on textures was 6.7 ± 3.4 (mean ± standard error) times greater than power at the resonance frequency during whisking in air (unpublished data).

Finally, we tested whether the amplitude of resonance frequency vibrations in each whisker depends on, and encodes, surface texture, as posited by the resonance hypothesis [11,13]. In isolated whiskers and anesthetized animals, prolonged, stable application of different texture or vibratory stimuli to the tip of a single whisker generates up to a 10-fold difference in steady-state power at the whisker's resonance frequency, indicating strong tuning for specific textures or vibration frequencies [11,22]. In contrast, we found that during natural whisking, the power spectrum for whisker vibrations in a single whisker was remarkably constant across different surfaces (e.g., the five sandpapers in Figure 7C and 7D). We calculated the power at the presumed resonance frequency as a function of texture for all sandpapers that were presented to each animal. Across the ten whiskers (rats N4–6, H1, and H2), no substantial or systematic relationship between sandpaper grade and vibration power at presumed resonance frequency was observed, either for nose poke or head-fixed rats (Figure 8B). On average, the maximal change in power at the presumed resonance frequency between any two textures for individual whiskers was 49 ± 29% (mean ± s.d.). This is substantially less than the 10-fold variation observed with prolonged, regular stimulation in anesthetized animals and detached whiskers. Two-way ANOVA found no significant differences in power at the presumed resonance frequencies in each behavioral trial for either whisker type (D1, D2, or D3) (*F*(2,1624) = 0.45; *p* = 0.64) or sandpaper grade (P150 through P1500) (*F*(6,1624) = 1.18; *p* = 0.32). Similar analysis of normalized power at the presumed resonance frequency (normalized to total spectral power, which controls for trial-to-trial variability in total vibration power) produced identical results (unpublished data).

**Figure 9:**
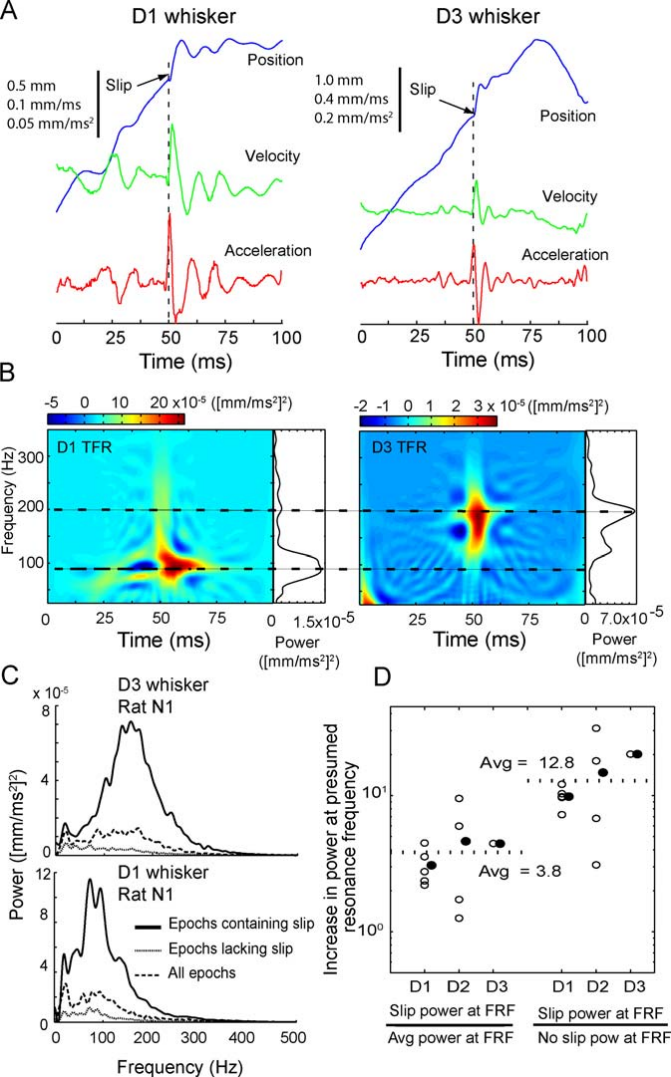
Resonance Vibrations on Surfaces Represent Transient Ringing Following Discrete High-Acceleration Events (A) Examples of high-acceleration whisker movement for a D1 and a D3 whisker in rat N1. Movement event onset is marked by an acceleration peak (aligned at 50 ms), followed by transient, decaying ringing in acceleration, velocity, and position. (B) TFR of the movement traces shown in (A), showing power at 90 Hz (D1 whisker) and 180 Hz (D3 whisker) following the high-acceleration event. (C) Comparison of average power spectra in 400-ms epochs containing a high-acceleration event (black), lacking such an event (dotted), or for all whisking epochs (dashed). Power spectra were derived from motion of the D1 and D3 whiskers in rat N1, averaged across all textures. (D) Analysis of increase in power at high-frequency (presumed resonance) peak during high-acceleration event, for all whiskers studied. Plot shows ratio of power at high-frequency peak in epochs containing high-acceleration events to power at this frequency for all whisking epochs (left), and to power at this frequency for epochs lacking high-acceleration events (right). Each open circle represents a single whisker averaged over all textures. Filled circles show average across all individual measured whiskers.

These results indicate that during active whisking under our experimental conditions, whiskers resonate on textures, but resonance magnitude is independent of texture roughness. This is contrary to the expectation of the resonance model for texture coding, which predicts that sustained whisker-tip movement over texture spatial features leads to regular whisker vibrations whose amplitude builds up most effectively when vibration frequency matches whisker resonance frequency [25]. One potential explanation of the present result is that resonance frequency vibrations do not build up in a gradual, sustained manner during natural whisking, but represent transient responses (ringing) to discrete high-acceleration, high-velocity events. Such events were a prominent feature of whisker movement across surfaces (e.g., Figure 7A and 7B), and were commonly associated with transient high-frequency ringing in whisker position, acceleration, and velocity. Representative examples of this behavior measured during protraction of D1 and D3 whiskers on a P150 sandpaper are shown in Figure 9A (see also Figure 7A and 7B). TFRs of these representative events revealed postevent ringing of the D1 whisker at approximately 90 Hz, and postevent ringing of the shorter D3 whisker at approximately 175 Hz (Figure 9B).

To determine whether transient ringing induced by these discrete motion events was a significant source of overall resonance vibrations during texture palpation, we compared vibration power spectra in 0.4-s epochs centered on high-acceleration events (defined here as movement events in which acceleration magnitude exceeded mean acceleration by 2 s.d.) versus equivalent epochs of whisker retraction or protraction when no high-acceleration event occurred. Results showed that power at the high-frequency peak was, on average, 12.8 ± 2.6 times greater in epochs containing high-acceleration events versus epochs that lacked such events, and 3.8 ± 0.8 times greater versus all whisking epochs, regardless of whether they contained an acceleration event (*n* = 10 whiskers, 5 animals). This result is shown for D1 and D3 whiskers of rat N1 in Figure 9C, and for all whiskers in Figure 9D. Together, these results demonstrate that resonance vibrations in whiskers during texture palpation primarily represent transient ringing following discrete high-acceleration movement events, and that the amplitude of resonance vibrations does not vary across the range of sandpapers that were tested.

### Characterization of Slip-Stick Events

**Figure 10:**
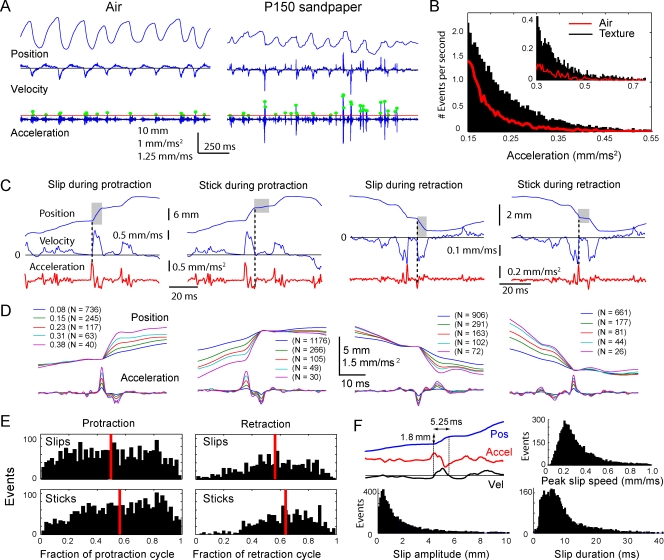
Characterization of Slips and Sticks (A) Example of the motion of the D2 whisker of rat H1 in air and on rough (P150) sandpaper. Dots indicate acceleration transients greater than 4 s.d. above mean acceleration in air (red line). (B) Frequency of different magnitude acceleration events during whisking in air (red line) and onto textures (P150, P400, P800, and P1200 combined) (bars) for the D2 whisker in rat H1. Inset, expanded view of high-acceleration events. (C) Example of slip and stick events (gray boxes) during whisker protraction and retraction on texture. (D) From left to right: mean slip during protraction, stick during protraction, slip during retraction, and stick during retraction, for D2 whisker in rat H1. Mean events were compiled separately for events with peak acceleration in the ranges of 0.08–0.15, 0.15–0.23, 0.23–0.31, and 0.31–0.38 mm/ms^2^ (legend indicates base of this range). (E) Number of slip and stick events during different phases of protraction and retraction (*n* = 10 whiskers, 5 rats: N1, N2, N3, H1, and H2). (F) Distribution of slip amplitude (net change in whisker position; lower left) slip duration (lower right), and peak speed (upper right), compiled across all slips for the ten whiskers in (E). Upper left, example of magnitude and duration measurement for one slip event. Duration was measured as the time from initial acceleration peak to return of whisker velocity to mean velocity.

Discrete high-acceleration motion events were prominent on textures, but were generally absent during whisking in air. Representative whisker motion in air and on a rough (P150) sandpaper are shown in Figure 10A. In this example, large-acceleration events (acceleration > 4 s.d. in air; green dots) occurred 2.5-fold more often on the sandpaper than in air. Acceleration events of all magnitudes occurred more frequently on the texture versus air for this whisker (Figure 10B, texture: 315 trials, 340 s of whisker-movement data; air: 111 trials, 223 s), and the highest acceleration events (>0.3 mm/ms^2^) were detected predominantly during whisking on texture (inset). Although we use acceleration as a convenient marker for these motion events, whisker acceleration and velocity were well correlated in whisker-motion traces (Figure S2).

High-acceleration events occurred during both protraction and retraction, and could be classified into slips (events in which whisker speed suddenly increased in the direction of whisker motion) and sticks (events in which speed suddenly decreased, corresponding to sudden stopping of whisker movement). Examples of slips and sticks during protraction and retraction are shown in Figure 10C. The average kinematics of slips and sticks during protraction and retraction are shown in Figure 10D, for the D2 whisker in rat H1 moving across four sandpapers. For each type of event, separate averages were calculated for five ranges of acceleration magnitude. (High-acceleration events correspond to more abrupt slips and sticks.) The average position and acceleration traces revealed that whisker slips were followed, on average, by sticks, and sticks were preceded by slips. Thus, sequences of high-acceleration events represented slip-stick motion of whiskers along surfaces.

Slips occurred during all phases of protraction and retraction (Figure 10E). To determine the average size and time course of a slip, we compiled histograms of slip magnitudes, durations, and peak speed (|velocity|), for all rats and all whiskers (*n* = 10 whiskers, 5 rats), including all slip events with acceleration greater than 2 s.d. of the acceleration in air (Figure 10F). Slip duration was defined from the initial acceleration peak to the time when whisker speed returned to the average speed. An example of the calculation of slip magnitude and duration is shown in Figure 10F (upper left). Results showed that during the average slip event, the whisker traveled a mean of 1.9 mm, in a mean of 8.6 ms, and achieved a peak speed of 0.33 ± 0.24 mm/ms, before whisker speed returned to average.

### Slip-Stick Events Encode Surface Texture

We tested whether slip-stick events could provide an alternate, nonresonance-based code for surface texture. A slip-stick code is plausible since sharp, high-acceleration, and high-velocity events effectively drive spikes in somatosensory cortex [7,26,27], and thus the pattern of slip-stick events is likely to be encoded in the rat's central nervous system (CNS). We again used acceleration to identify these events. We compared acceleration events on four sandpaper textures (P150 [very rough], P400, P800, and P1200 [very smooth]) that were interleaved in blocks for each rat within a single day (five or ten trials per block). This measurement was performed for the D1 and D2 whiskers in three rats (N6: 89–103 trials per texture, H1: 52–56 trials per texture, and H2: 40–43 trials per texture). Analysis was restricted to within-day comparisons across textures to avoid complications from day-to-day variability in whisking behavior. For this analysis, an acceleration event was defined as any acceleration peak that crossed a defined threshold, with a minimum of 2 ms between events, and stick versus slip events were not distinguished.

**Figure 11:**
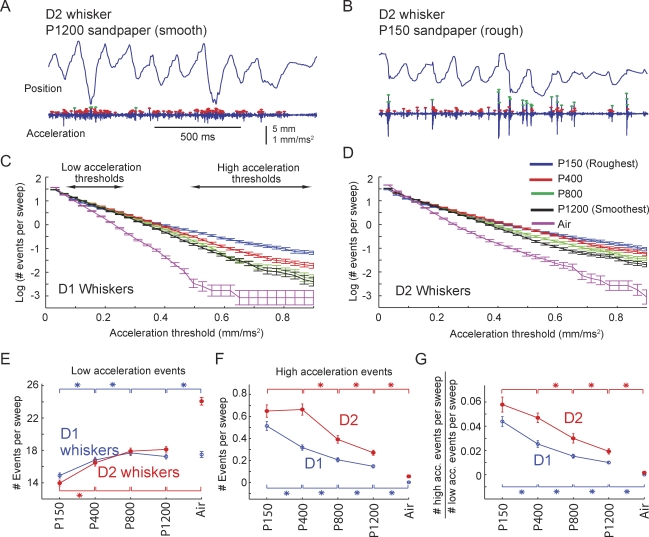
Relationship between High-Acceleration Slip/Stick Events and Texture (A and B) Representative motion of the D2 whisker on P1200 (smooth) versus P150 (rough) sandpaper in rat H2 (same whisker as in Figure 10A). Red dots mark low-acceleration events (0.062–0.248 mm/ms^2^); green dots mark high-acceleration events (>0.496 mm/ms^2^). (C and D) Mean number of acceleration events per sweep (protraction or retraction movement) during whisking in air and on four sandpapers, calculated across three D1 whiskers and three D2 whiskers in three rats. Number of events per sweep is plotted on a log scale. Each point shows the cumulative number of events with acceleration greater than the threshold indicated on the *x*-axis. Error bars represent the standard error of the number of acceleration events per sweep (*n* = 644–1,247 sweeps per texture). (E) Number of low-acceleration events (events with acceleration in the range 0.062–0.248 mm/ms^2^, corresponding to 1–4 s.d. above zero) measured on four textures and air. Asterisks indicate significantly different numbers of events between the indicated textures, for the D1 whisker (blue) and the D2 whisker (red) (Mann-Whitney *U*-test, *p* < 0.01). (F) Number of high-acceleration events (>0.496 mm/ms^2^, 8 s.d. above zero) across four textures and air. (G) Ratio of the number of high- to low-acceleration events per sweep, as a function of texture roughness. Data shown in (E–G) are from three D1 whiskers and three D2 whiskers; error bars are the standard error bars calculated for the number of events per sweep, as in (C) and (D).

Motion of the D2 whisker in rat H2 across a smooth (P1200) and rough (P150) sandpaper is shown in Figure 11A and 11B. (This is the same whisker whose motion in air and on P150 sandpaper was shown in Figure 10A.) Low-acceleration events (red dots, peak acceleration 0.062–0.248 mm/ms^2^, corresponding to 1–4 s.d. above zero on the P1200 surface) occurred on both textures, as well as in air. In contrast, high-acceleration events (green dots, >0.496 mm/ms^2^, corresponding to 8 s.d. above zero on the P1200 surface) occurred preferentially on the rough P150 sandpaper. This suggested that high-acceleration events may occur systematically more frequently on rougher surfaces.

We calculated the average incidence of different magnitude acceleration events on P150, P400, P800, and P1200 textures, as well as during whisking in air, for six whiskers in three rats (rat N6 performing the nose poke task, and rats H1 and H2 whisking while head-fixed; D1 and D2 whisker motion was measured in each animal) (Figure 11C and 11D). The number of acceleration events surpassing different absolute acceleration thresholds was calculated per sweep, where a sweep was defined as a single whisker protraction or retraction. Results showed that the total number of acceleration events surpassing low acceleration thresholds (e.g., 0.1 mm/ms^2^) was not different between whisking in air and whisking on surfaces, but the number of events surpassing high acceleration thresholds (e.g., 0.4 mm/ms^2^) was higher on surfaces than in air, and was systematically higher on rougher versus smoother surfaces (Figure 11C and 11D). Statistical analysis showed that low-acceleration events (with peak amplitude in the range 0.062–0.248 mm/ms^2^, corresponding to 1–4 s.d. above zero) were equally prevalent in air and on smooth P1200 and P800 surfaces, but were significantly less prevalent (asterisks; Mann-Whitney *U*-test, *p* < 0.01) on the rougher P400 and P150 surfaces, especially for the D2 whisker (Figure 11E). Conversely, high-acceleration events (>0.496 mm/ms^2^, corresponding to 8 s.d. above zero) were systematically more prevalent on rougher versus smoother surfaces, for both D1 and D2 whiskers (Figure 11F). As a result, the ratio of high to low acceleration events per sweep increased systematically and significantly with surface roughness (Figure 11G; asterisks indicate significant differences in ratio between pairs of textures). These relationships between slip-acceleration magnitude/frequency and surface roughness held true for both the nose poke rat (N6) and head-fixed whisking rats (H1 and H2) (unpublished data). These results suggest that either the frequency of high-acceleration events or the relative frequency of high to low acceleration events may contribute to a kinetic signature for surface roughness [7], independent of whisker resonance.

## Discussion

Sensory systems generate signals by physical interaction between sensory organs and the external environment, and the form of this interaction determines how features of the sensory environment are encoded. In the whisker system, active movement of the whiskers and whisker mechanical properties critically determine this interaction, and therefore influence neural coding [6,28]. We attempted to distinguish between two major models of whisker texture coding—the resonance hypothesis [11–13,22] and the kinetic signature hypothesis [7,10,29]. These hypotheses assume different physical interactions between whiskers and objects, different patterns of surface-induced whisker vibration, and different neural coding strategies [6]. In the resonance hypothesis, each whisker is tuned to resonate most strongly in response to a specific range of textures (those textures that drive tip vibration at the whisker's intrinsic resonance frequency). Because resonance frequency varies with whisker length, texture information is encoded spatially by the relative amplitude of resonance vibrations across whiskers, and in somatosensory cortex (S1) by relative firing rates of neurons across whisker columns [11–13,22]. In the kinetic signature hypothesis, mechanical resonance plays no special role in coding. Instead, textures generate unique, identifiable motion patterns in single whiskers, and texture information is encoded in the brain by neuronal spiking that tracks features of these patterns, including mean speed of whisker vibration [10] and irregular whisker-velocity patterns [7], which vary with texture [7].

We tested these hypotheses by measuring the physical vibrations induced in whiskers as rats actively whisked in air and across textured surfaces. Whiskers were found to resonate in air and on textures, but the amplitude of resonance vibration was equal across a wide range of textures. Thus the resonance hypothesis of texture coding is not correct, at least for the behavioral conditions and range of textures tested here. Instead, we found whiskers exhibited discrete, high-acceleration, high-velocity slip-stick events on surfaces that drove transient ringing in the whiskers. We propose that slip-stick (or slip-stick-ring) events are fundamental elements of natural whisker–surface interaction. Because the rate and magnitude of slip-stick events were correlated with texture, we propose that slip-stick events may contribute to a unique kinetic signature for textures in individual whiskers.

### Evaluation of the Resonance Hypothesis for Texture Coding

We found that whiskers exhibited high-frequency (>20 Hz) vibrations during active whisking in air, and that the spectral composition of these vibrations varied with whisker length, due to filtering by whisker resonance (Figures 2–5). Thus, whiskers resonate during natural whisking in air, and a map of whisker resonance exists in awake, whisking rats (Figure 4). High-frequency vibrations were coherent across neighboring whiskers, were not caused by head motion or interactions between whisker and air (Figure 2C–2E), and could be elicited by high-frequency stimulation of the facial nerve in anesthetized animals (Figure S1). This suggests that vibrations are due to neurally or mechanically coordinated drive of neighboring whiskers by whisker facial muscles. EMG recordings of extrinsic and intrinsic muscles detected high-frequency components of muscle contraction. However, when we measured spectral coherence between whisker vibrations and EMG activity, we found only modest coherence for frequencies up to approximately 50 Hz (near the FRF of arc 1 whiskers), and nonsignificant coherence for frequencies greater than 50 Hz (near the FRF of arc 2 and shorter whiskers) (Figure 6). This suggests either that (1) additional coherent, high-frequency muscular drive exists, but was not detected by the EMG recordings, or that (2) muscles drive resonance vibrations noncoherently, as could occur if sharp, pulsatile muscle contractions induced higher frequency vibrations and excited whisker ringing at the resonance frequency. This latter case is less likely because whisker motion, and muscle drive, are relatively smooth during exploratory whisking. However, sharp contractions may occur during more erratic whisker motion.

Resonance vibrations also occurred during active whisking on sandpaper surfaces, as inferred from the presence of spectral peaks in whisker vibration at specific supra-whisking frequencies, with longer whiskers vibrating at low frequencies, and shorter whiskers vibrating at higher frequencies (Figures 7 and 8). Thus, resonance filters whisker vibrations during whisking onto surfaces. However, resonance vibrations occurred primarily as transient, sporadic ringing events, rather than as sustained oscillation, and neither the amplitude of vibrations at presumed resonance frequencies nor the overall power spectrum varied with texture across a wide range of sandpaper grades (Figures 7 and 8). Thus, each whisker was not preferentially excited by a specific set of textures. We conclude that differences between sandpaper textures are not encoded by relative vibration amplitude across facial whiskers, at least in the geometrical and behavioral conditions of our study. These data argue against the resonance hypothesis for texture coding. However, they do demonstrate that whisker resonance occurs during surface palpation, and therefore may play a role in amplifying some types of whisker responses [13]. These results confirm a recent study that detected resonance vibrations on textured surfaces, but did not examine whether resonance encoded texture [17].

The critical difference between our results and the resonance hypothesis appears to be in how resonance vibrations are generated during whisker–surface interaction. Linear resonating systems can resonate in two distinct modes: In the transient mode, oscillations are triggered by discrete external impulses, and occur transiently after these impulses, in the absence of additional external vibratory forces. In this case, oscillation dynamics are determined solely by the intrinsic properties of the system, as in the case of transient resonant ringing of a tuning fork after being struck by an object. In the steady-state mode, in contrast, vibrations are produced in an ongoing manner during sustained external vibratory drive. In this case, vibratory responses occur at the same frequency as the external impulses, and vibration amplitude is much larger when external vibrations occur at the intrinsic resonance frequency of the system. The resonance hypothesis assumes that passage of a whisker over a surface generates sustained tip vibrations as the whisker interacts with surface microfeatures, and that this causes steady-state resonance to build up in the whisker. Such steady-state resonance indeed occurs when sustained vibrations are applied to isolated whiskers or to nonmoving whiskers in anesthetized animals [11,12,22]. However, our results demonstrate that voluntary whisker motion produces discrete, high-acceleration slip-stick events, rather than smooth motion across surfaces (Figure 10). These slip-stick events drive transient ringing, and this transient ringing is the major source of whisker resonance on surfaces (Figure 9). The dominance of transient resonance, as opposed to sustained resonance, explains why whisker vibrations vary with intrinsic properties of the whiskers (Figure 8A), but not with surface texture (Figure 8B). These results confirm a previous observation that sustained resonance vibrations do not appear during voluntary whisking on surfaces [12].

Together, these data indicate that whisker resonance occurs in awake animals, both during whisking in air and on surfaces, and may contribute to encoding or amplification of certain aspects of whisker input. However, differential whisker resonance does not encode texture in these behavioral conditions and using these sandpaper surfaces, which are predicted to be discriminable by rats [4,5,10]. We cannot rule out that, under conditions of behavioral discrimination, rats may adopt a different whisker exploration strategy that may enable resonance-based coding of texture. However, recent studies of texture discrimination have provided no evidence for coding by resonance [10,17], and two arguments suggest that such a coding strategy may be problematic: first, the relationship between whisker resonance frequency and effective whisker length (Figure 8A) suggests that any trial-to-trial variation in surface position or angle relative to the face will alter whisker resonance frequency, making it difficult to construct a position-independent resonance code for texture. Second, rats discriminate textures even with substantial trial-to-trial variation in whisking speed [5]. Such variation will alter the relationship between texture spatial frequency and whisker-tip vibration frequency, making it unlikely that a whisker could be “tuned” for a specific texture.

### Slip-Stick Events as Elemental Units of Whisker–Surface Interaction

A common feature of whisker motion across sandpapers were discrete, high-acceleration slip and stick events (Figure 10). Slip and stick events occurred during all phases of whisker protraction and retraction (Figure 10E). These events often generated high-amplitude transient ringing at the whisker's resonance frequency (Figures 7A and 9). Slip-stick events were frequent: for example, 1.2 events with acceleration greater than 0.4 mm/ms^2^ occurred per protraction–retraction cycle for the D1 whisker, averaged across all sandpaper surfaces (Figure 11C). This corresponds to approximately 30 events when all 25 large whiskers on each side of the face are considered. These events have also been observed during whisking onto surfaces under very different geometrical and behavioral conditions [17], and are therefore likely to be basic common elements of the whisker input stream.

The average slip was 1.9 mm (measured at the whisker midpoint, ∼10 mm from the follicle), and lasted 8.6 ms before whisker velocity returned to its mean value (Figure 10F). This corresponds to a mean angular displacement of 10° and a mean velocity of 1,100°/s during slips. Peak velocity during slips was 0.33 mm/ms. This amplitude and velocity are well within the range of behavioral detectability [30] and spike encoding at primary afferent and cortical levels [26,27,30]. Thus, slip-stick events are likely to be encoded in the CNS. These slip-stick events are similar to velocity transients observed during artificial whisking onto textures in anesthetized rats [7,15]. Because high-acceleration events occur more frequently on textures than in air (Figure 11), we propose that slip-stick events may encode the presence of a surface, or surface properties, on the whisker array.

### Slip-Stick Events as a Component of the Kinetic Signature for Texture

The kinetic signature hypothesis for texture coding proposes that textures generate unique, identifiable temporal patterns of whisker vibration (“kinetic signatures”) in single whiskers, and that these temporal features are encoded in neural spike trains. Candidate components of kinetic signatures for texture include the spectral composition of whisker vibration [15], the mean speed of whisker vibration [7,29], and the temporal profile of velocity transients [7]. These features vary when whiskers of anesthetized rats are artificially swept across different textures by electrical stimulation of the facial motor nerve, with rough versus perfectly smooth textures generating differences in mean vibration speed [7,29], and finer texture differences (e.g., between sandpaper grades) generating unique temporal profiles of whisker velocity [7].

Our data suggest that slip-stick events may contribute to the kinetic signature for texture. The magnitude and frequency of these events were correlated with texture, with rougher sandpapers eliciting a greater frequency of high-acceleration events (which tend to also be high-velocity events), and a higher proportion of high-acceleration versus low-acceleration events, compared to smoother sandpapers and to air (Figure 11). This relationship between slip acceleration and texture is expected from a simple model in which rougher surfaces, which have greater friction, require more forward force during whisker protraction (or retraction) to overcome static friction and move the whisker tip forward (or back). This increased forward force translates into increased acceleration during forward slips. Thus, more high-acceleration slips, and fewer low-acceleration slips, are predicted on rougher textures. This significantly extends a prior study showing more high-speed slip events on a rough surface versus a completely smooth one [17].

We propose that slip magnitude (acceleration or velocity) and frequency are components of the kinetic signature for texture in the whiskers, and that coding of these parameters by S1 neurons provides information about surface texture. In anesthetized animals, whisker deflections evoke phasic, single-spike responses in S1 neurons, with spiking probability positively correlated with whisker velocity and acceleration over the ranges of 0.02–1.0 mm/ms [31,32] and approximately 20–500 m/s^2^ [33], respectively. The range of slip speeds and accelerations observed here (∼0.1–0.5 mm/ms and ∼100–1,000 m/s^2^) fall within this dynamic range. Thus, the occurrence and magnitude of slips are likely to be encoded by time-locked spikes in S1 ensembles, with texture-related sequences of slip-stick events (Figure 10A) encoded by temporal sequences of spikes (constrained by the intrinsic dynamics of whisker circuits and synapses). The occurrence of discrete slip events related to texture, observed here under two behavioral conditions, suggests a potential temporal spike code for texture during awake, active sensation. Such a temporal code has been suggested from S1 recordings in anesthetized rats during electrically evoked whisking on texturally similar surfaces, like the sandpapers used here [7]. In contrast, active whisking onto very distinct textures (rough vs. smooth glass) evokes subtly, but significantly different, mean firing rates in S1 [10]. Slip-evoked spikes could drive such texture-specific changes in firing rate, depending on neural sensitivity to slip amplitude and velocity.

## Materials and Methods

Ten rats were used in this study. All procedures were approved by the University of California San Diego (UCSD) Institutional Animal Care and Use Committee and followed Society for Neuroscience guidelines for research.

### Behavioral training.

Two types of whisker behavior were studied. In Behavior 1 (whisking in nose poke, six rats), rats were trained using operant conditioning techniques to place their nose in a small port (the nose poke) and whisk for approximately 1 s in air or on textured surfaces. The behavioral apparatus, modeled after [34], consisted of an outer reward chamber containing a solenoid-gated drink port, and an inner measurement chamber containing the nose poke, texture stimuli, and whisker-motion recording system (Figure 1A). Rats received water (50 μl) as reward during behavioral training (1 h per day) and during a 1-h ad lib drinking period following each behavioral training session, but not during the remaining 22 h per day, 5 d per week. Water was freely available on weekends. Rats on this regimen were healthy and alert, and gained weight daily.

Rats (age 30 d) were initially accommodated to handling (3–5 d) and to the behavioral apparatus. Rats were then trained to drink from the drink port in response to a white noise tone (WNT). A phototransistor in the drink port signaled the rat's presence and gated water delivery. Next, rats were trained to nose poke to trigger the WNT and water delivery to the drink port. A phototransistor in the nose poke reported nose poke occupancy. Finally, rats were trained to gradually increase nose poke duration and to actively whisk while in the nose poke. Gross whisking was assessed by four phototransistors that generated voltage pulses when the whiskers passed over them. The number of phototransistor pulses required to trigger the WNT and drink port water delivery was gradually increased until rats were whisking in the nose poke for approximately 0.5 s. Each approximately 0.5-s bout of whisking in the nose poke was considered a trial, and trials were separated by the rat retreating to the reward chamber to drink. Trained rats performed 80–150 trials per day. Total training time (after accommodation) was approximately 23 d.

Whisker motion was recorded optically in trained rats whisking in air and whisking onto textures. Textures were 6 × 6-cm sandpapers of grade P150, P240, P400, P600, P800, P1200, and P1500 glued to an aluminum plate and positioned in the whisking path of the right whiskers 5 mm from the whisker tips, parallel to the face. Up to four different textures were mounted on a four-arm Plexiglas holder attached to a stepper motor (Oriental Motor, PK264B1A-SG10). Textures were rotated into place between trials while the rat was at the drink port. Surface positioning relative to the nose poke was performed as follows: first, using videography, we measured the mean position and orientation of the external edge of the whisker pad while the rat was performing the whisking behavior. Surface orientation was set parallel to the whisker pad. Next, we transiently anesthetized the rat and measured the length of the whisker to be studied (whisker movement on surfaces was measured for a single whisker at a time). We positioned the stepper motor so that the point on the surface closest to the face (i.e., the point at the intersection of the surface and of the whisker, when the whisker was normal to the face) was located 5 mm closer to the whisker pad than the whisker length. Surface positioning was verified by imaging the whisker 0.5 mm from the surface, and confirming that the whisker shadow disappeared from the imaging plane when the surface was moved approximately 5 mm from its set position. Whisking in air was measured by rotating the stepper motor into a position with no texture present. Thus, up to four textures (or three textures plus air) could be interleaved under computer control during a recording session. Training and recording procedures were controlled by custom routines in Labview (National Instruments).

In Behavior 2 (whisking while head-fixed, four rats), rats (age 30–40 d) were accommodated to handling (∼1 wk), and to being placed for 15 min in a loose fabric sack from which the head emerged (∼1 wk) [35]. Rats were habituated to being placed, while in the sack, in a 5-cm–diameter Plexiglas tube (Figure 1B). Rats then underwent surgery to implant electromyogram (EMG) recording electrodes (see below), during which a small screw was affixed to the skull with dental acrylic. After 4–6-d recovery from surgery, rats were placed again in the Plexiglas tube, and the head was stabilized via the screw (Figure 1B). Head-fixed rats naturally whisked in response to objects held in front of them. Whisker motion was measured during these whisking epochs. Recording sessions typically lasted 15–30 min. Whisker motion was recorded in 3-s trials with approximately 50–100 trials per recording session. The animal was positioned so that the head and whiskers were in the same spatial relationship to the textures and CCD imaging array as in Behavior 1. Textures (or air) were presented in blocks.

For both Behaviors 1 and 2, behavioral training was performed with all whiskers intact. The day before whisker-motion measurement, rats were transiently anesthetized with isoflurane, and all whiskers whose motion was not being studied were trimmed at the base. For Behavior 1, all but one to four whiskers (δ, D1, D2, and D3) were trimmed. For Behavior 2, all but two to three whiskers in the C row or D row were trimmed.

### Measurement of whisker motion.

Whisker motion was measured in one dimension by casting shadows of the whiskers onto a linear CCD imaging array. The light source was a diode laser (670 nm), positioned above the rat and focused into a collimated line 60-mm long and 1-mm wide, using two cylindrical lenses rotated 90° from one another (Figure 1C). Below the whiskers, a third cylindrical lens focused whisker shadows onto the linear CCD array (Fairchild imaging, CCD 133AEDC, 1,060 elements, 13-μm width per element). The output of every other CCD element was sampled at 4-kHz frame rate using custom-built electronics (UCSD Physics electronics shop) and a National Instruments data acquisition card (PCI 6111). Voltage traces from the array were stored and processed offline to determine whisker position. In Behavior 1, whisker position was recorded for 1.5 s starting with nose poke onset (analysis was restricted to the epoch during which the rat remained in the nose poke). In Behavior 2, whisker motion was recorded in 3-s blocks.

The CCD array was positioned parallel to the whisker pad, either approximately 10 mm (range: 6–14 mm) from the whisker pad (whisking in air) or at the midpoint between the texture and the whisker pad (whisking on texture). Whisker contact with textures was verified for each rat by the consistent presence of whisker shadows when the array was positioned 1 mm from the texture. Each frame of CCD output was subtracted from a baseline CCD image obtained when no whiskers were present (baseline images were obtained several times during each recording session). Whisker shadows appeared as discrete voltage peaks in the baseline-subtracted CCD image, with each shadow covering eight to ten CCD pixels. Position of each whisker shadow was calculated as the weighted mean of all pixels in the whisker shadow, weighted by pixel voltage. Whisker spatial position was calculated from whisker-shadow pixel position via a calibration curve obtained using a 0.5-mm spaced wire grid held at whisker position. Repeated measurements showed that whisker position was determined with a spatial resolution of approximately 5 μm.

Whisker motion over time was computed algorithmically using custom software in Matlab. Up to four whisker shadows could be tracked simultaneously and identified unambiguously using this method. All but the imaged whiskers were trimmed weekly to the level of the skin, during transient isoflurane anesthesia (4% in 2 l/min O_2_, delivered via a nose cone). If a whisker transiently left the imaging plane, whisker motion was only analyzed up to that point.

### Fundamental resonance frequency measurement.

To measure whisker FRF using the impulse method, rats were anesthetized with isoflurane and the head positioned in the behavioral apparatus at the standard position and angle relative to the CCD array. An impulse was delivered manually to each whisker, and the resulting decaying oscillation was measured with the CCD array. The FRF was calculated as the inverse of the average time between peaks in the oscillations [11]. For untrimmed whiskers, the first and second harmonics of the resonance frequency were calculated as: *f*_1_ = (10.6/4.4)FRF and *f*_2_ = (19.2/4.4)FRF, as predicted by theoretical models of tapered beams [23]. For trimmed whiskers, which are truncated tapered beams, the ratios *f*_1_/FRF and *f*_2_/FRF are functions of the truncated length. We calculated *f*_1_ and *f*_2_ for trimmed whiskers by interpolating *f*_1_/FRF and *f*_2_/FRF ratios that were numerically calculated by Conway et al. [18] for four different ratios of truncated to untruncated length of a thin conical beam.

Whisker length was measured while rats were anesthetized. Length was measured from the skin surface to the whisker tip, using calipers and 4× magnification under a dissecting microscope.

### EMG recording.

In some experiments, EMG activity was recorded from whisker pad muscles. EMG electrode implantation followed Berg and Kleinfeld [21]. Briefly, surgery was performed using sterile technique, under ketamine/xylazine anesthesia (90 and 10 mg/kg, respectively, i.p.). Supplemental ketamine (20 mg/kg) was administered approximately every 2 h to maintain anesthetic depth, determined by absence of limb withdrawal reflex and breathing rate of 45–60 breaths per min. EMG electrodes were made from Teflon-coated tungsten microwire (0.002” diameter; California Fine Wire; 1 mm of insulation stripped at recording tip). Microwires were implanted in pairs to record the differential EMG signal. Microwires were implanted via a midline incision at the top of the skull, and a lateral incision caudal to the mystacial pad. One electrode pair was implanted in the extrinsic muscle *m. nasolabialis*, by exposing this muscle and pressing the recording tips into muscle tissue. Microwires were secured at the muscle entry point using 6–0 Ethicon nylon sutures (Johnson and Johnson). To record EMG in intrinsic muscles, microwire pairs were threaded through a 26-ga targeting needle, which was used to insert wire tips into the whisker pad [21]. Wire tips were bent back at the needle tip to anchor the wires to whisker pad tissue. Wires were sutured in place where they exited the pad. Reference wires (stripped of 4 mm of insulation) were implanted in the dermis at the tip of the snout, rostral of *m. transversus nasi*. Microwire tip position was verified at the end of surgery by passing current to stimulate the muscles and evoke appropriate whisker and pad movements. Microwires were soldered into a ten-pin connector (Samtec) attached to the skull. Bupivicaine (0.1 ml) was administered for postoperative analgesia.

EMG recording commenced 4–6 d after EMG implantation. EMG data were collected in behaving rats in 3-s–long blocks, simultaneous with whisker-motion data. EMG signals were amplified (20× gain) and impedance buffered using an eight-channel head-mounted headstage amplifier (Plexon Instruments HST/8o50-G20). Headstage output was transmitted via twisted thin-gauge wires to a second amplifier and bandpass filter (Plexon Instruments PBX2/16sp-G50) (50× gain, 0.3–8 kHz bandpass). Amplifier output was digitized at 32 kHz (National Instruments PCI 6259). Analysis was performed on rectified, low-pass filtered (500 Hz cutoff) difference of neighboring raw EMG signals, denoted |∇EMG|. EMG and whisker data acquisition were performed on separate, synchronized data acquisition cards.

### Power spectra and coherence estimation.

Power spectra of whisker motion in air were calculated using the multitaper estimation technique of Thomson (1982) [36]. Briefly, a whisker motion time series recorded during a single trial was first multiplied by a set of *K* orthogonal data tapers. The Fourier transform of each tapered time series was calculated using the Fast Fourier Transform algorithm in Matlab, and from each Fourier Transform, the power spectrum was estimated as the modulus squared of the Fourier Transform. The estimated power spectrum of the whisker motion for the *n* ^th^ trial, 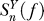 Z, was then an average over these *K* power spectra

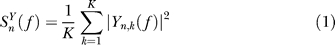
where *Y_n,k_* (*f*) is the Fourier Transform of the *k* ^th^ tapered time series of the *n* ^th^ trial. The average power spectrum of whisker motion for a single day's recording session was then an average over all trials performed on that day:

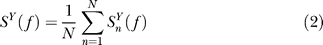
where *S^Y^* (*f*) is the average power spectrum of the whisker motion and *N* is the total number of trials. Here, *N* was typically between 50 and 150 trials, and the number of tapers, *K*, was 5. With this method, the resulting average power spectrum of a time series of duration *T* is smoothed over a half-bandwidth of

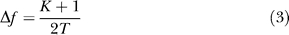



The spectral coherence *C*(*f*) between the |∇EMG| and whisker motion was calculated similarly to [37],

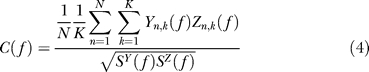
where *Z* (*f*) is the Fourier Transform of the |∇EMG| time series and *S^Z^* (*f*) is the average |∇EMG| power spectrum. The theoretical confidence intervals for coherence were computed following [38], where it is estimated that the coherence magnitude will exceed 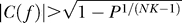 in *P* × 100% of measurements. Here, we take *p* = 0.05. Spectral estimation was performed using the Chronux (http://www.chronux.org) and signal processing toolboxes in Matlab.

### Wigner-Ville time-frequency representation.

The Fourier methods described above are appropriate for describing the average spectral properties of stationary signals [38]. We used the Wigner-Ville TFR to examine the brief transient ringing events during whisking onto textures. The TFR is known to provide good localization in both time and frequency, and is better suited for analyzing time series with time-varying frequencies [39]. This distribution is computed by correlating the entire time series *y*(*t*), with a time-translated version of itself and taking the Fourier transform of this locally autocorrelated function,

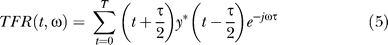



The color plots of the TFR were smoothed over time (windowsize = 2.5 ms) and frequency (windowsize = 20 Hz) for visualization. Power spectra of whisker motion onto textures were calculated by numerically integrating the unsmoothed *TFR*(*t*,*ω*) over time [39].

### Artificial whisking methods (for experiments shown in Figure S1).

In artificial whisking experiments [40], an incision was made in the side of the snout posterior to the whisker pad of the anesthetized animal (urethane 1.5 g/kg). The buccal motor nerve was separated from the underlying muscle and cut to prevent antidromic activation of the motor nerve [19]. The lower branch of the buccal nerve was also cut, which generated more-natural, horizontal whisks than with this nerve intact. The distal portion of the facial nerve was isolated in a stimulating cuff with electrodes placed around the nerve. Saline was applied to keep the nerve moist. The nerve was stimulated with brief monophasic pulses (50-μs duration, 3–6 V) from a Grass Stimulator (Model S88K). Pulses were generated at 110 Hz in bursts lasting 50 ms followed by 50 ms with no stimulation. Whiskers protracted during the bursts and passively retracted during the 50 ms following the bursts, generating 10-Hz artificial whisking.

## Supporting Information

Figure S1 Electrical Stimulation of Facial Motor Nerve Evokes High-Frequency Whisker Movements and Drives Whisker Resonance in Anesthetized Animals (A) Position of D1 and D2 whiskers, measured simultaneously, in response to 110-Hz burst stimulation of the facial nerve. Nerve stimulation evoked high-frequency, approximately 110-Hz whisker vibrations superimposed on slower artificial whisking motion. The amplitude of high-frequency vibrations was increased when the D1 whisker was progressively trimmed to bring whisker resonance frequency near the 110-Hz driving frequency (center panel). Whisker resonance frequency was measured at each whisker length, by the impulse method.
(B) Power spectrum analysis of the same experiment as in (A), showing data for all D1 whisker lengths that were measured. The D2 whisker was untrimmed throughout. Results show that whisker vibrations were maximally amplified when the FRF intersected with the 110-Hz drive frequency (shown by black arrow; white circles, asterisks, and diamonds represent the FRF and the first and second harmonics of the FRF, respectively).
(C) Same experiment in a second rat in which D2 was progressively trimmed, and D3 remained intact. Amplification of whisker vibrations occurred when FRF matched the drive frequency (left panel), or when the first harmonic of the resonance frequency matched the first harmonic of the drive frequency (left and right panels).
(646 KB PDF)

Figure S2 Correlation between Whisker Acceleration and Velocity during Slip-Stick Motion Events on Surfaces Peak velocity of slip-stick events is highly correlated with peak acceleration (*r* = 0.76 [4,649]; p < 0.01). Slip-stick events from all ten whiskers on all textures were identified by acceleration peaks with magnitude greater than 0.15 mm/ms^2^. Peak velocity was defined as the maximum velocity in a 10-ms window centered on the acceleration peak. Best-fit line is shown in red.
(464 KB PDF)

## Abbreviations

CCD: charge-coupled device
EMG: electromyogram
FRF: fundamental resonance frequency
TFR: time-frequency representation
