# Supplementary figures and images for: Texture Coding in the Rat Whisker System: Slip-Stick Versus Differential Resonance
Source: PLoS Biol. 2008 Aug 26;6(8):e215. doi: 10.1371/journal.pbio.0060215 (PMC2525689; doi:10.1371/journal.pbio.0060215)

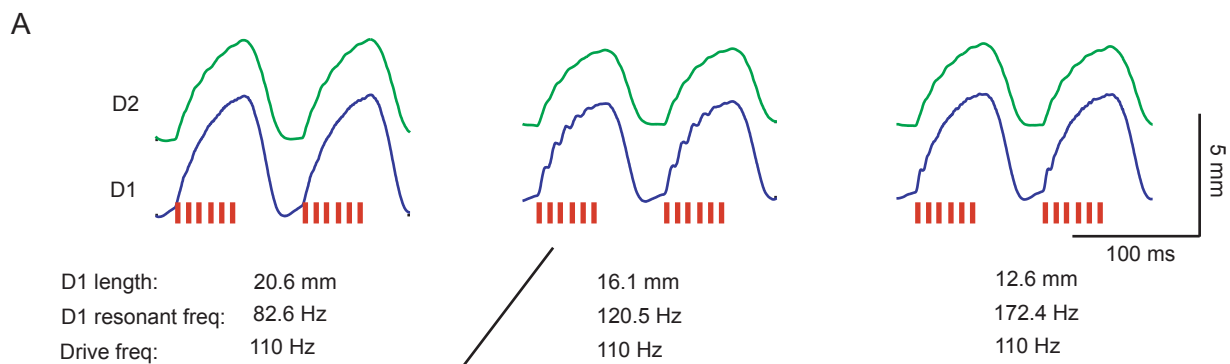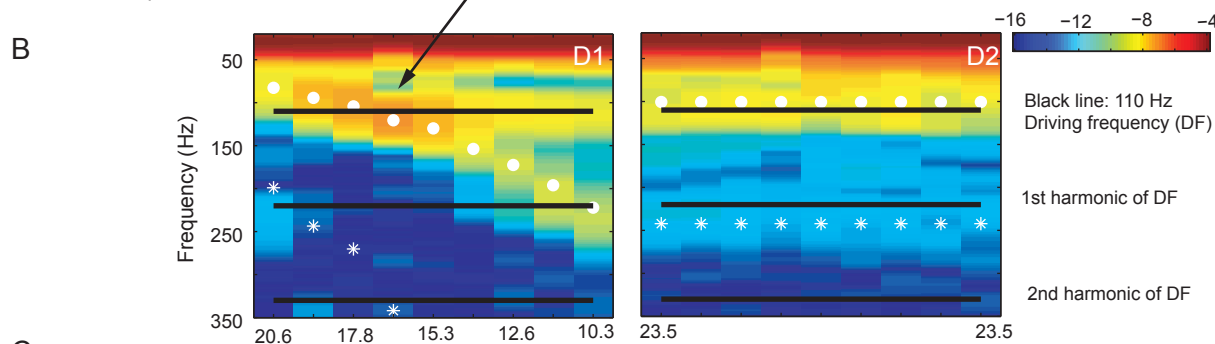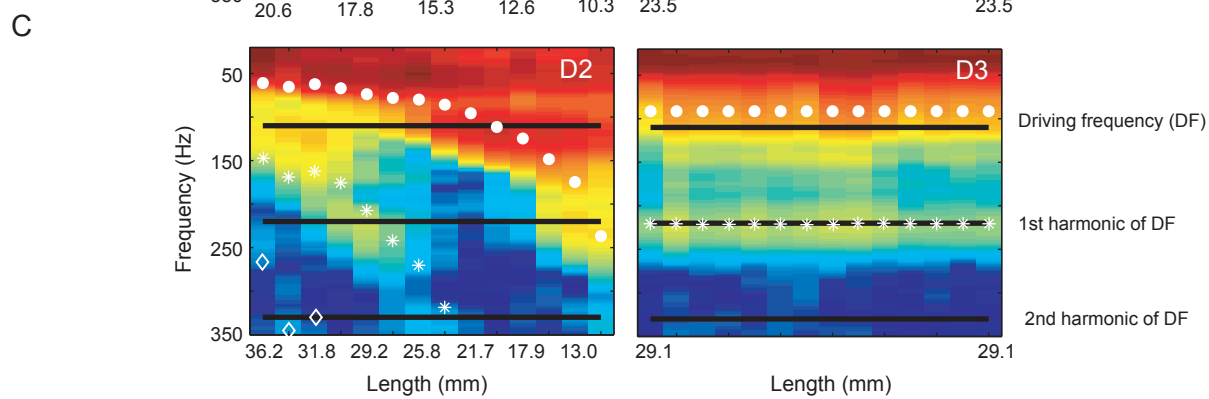

Supplement: Figure S1 — (A) Position of D1 and D2 whiskers, measured simultaneously, in response to 110-Hz burst stimulation of the facial nerve. Nerve stimulation evoked high-frequency, approximately 110-Hz whisker vibrations superimposed on slower artificial whisking motion. The amplitude of high-frequency vibrations was increased when the D1 whisker was progressively trimmed to bring whisker resonance frequency near the 110-Hz driving frequency (center panel). Whisker resonance frequency was measured at each whisker length, by the impulse method. (B) Power spectrum analysis of the same experiment as in (A), showing data for all D1 whisker lengths that were measured. The D2 whisker was untrimmed throughout. Results show that whisker vibrations were maximally amplified when the FRF intersected with the 110-Hz drive frequency (shown by black arrow; white circles, asterisks, and diamonds represent the FRF and the first and second harmonics of the FRF, respectively). (C) Same experiment in a second rat in which D2 was progressively trimmed, and D3 remained intact. Amplification of whisker vibrations occurred when FRF matched the drive frequency (left panel), or when the first harmonic of the resonance frequency matched the first harmonic of the drive frequency (left and right panels). (646 KB PDF) [file pbio.0060215.sg001.pdf]

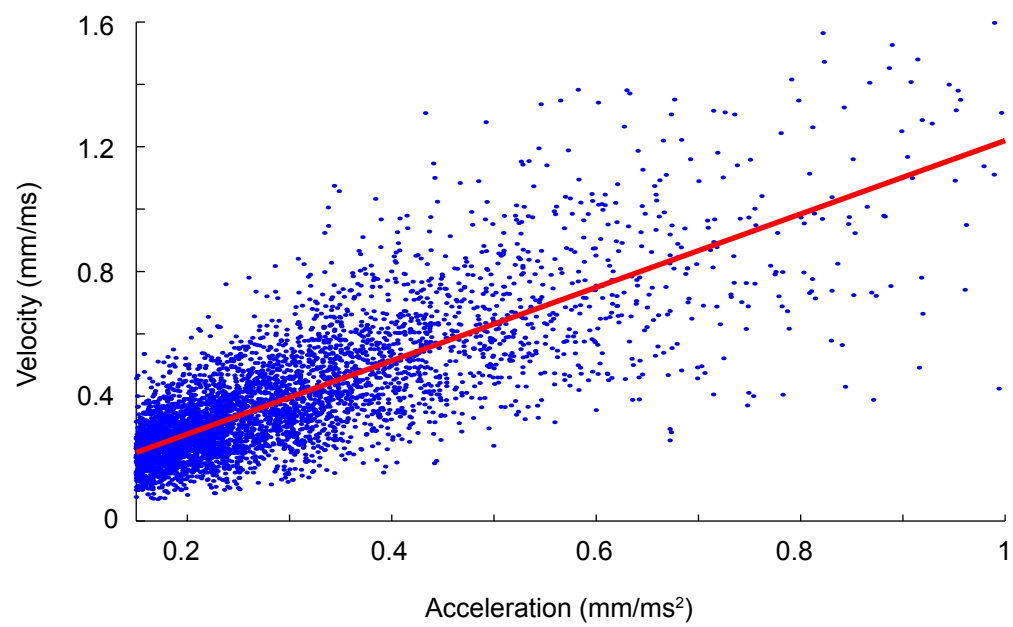

Supplement: Figure S2 — Peak velocity of slip-stick events is highly correlated with peak acceleration (r = 0.76 [4,649]; p < 0.01). Slip-stick events from all ten whiskers on all textures were identified by acceleration peaks with magnitude greater than 0.15 mm/ms2. Peak velocity was defined as the maximum velocity in a 10-ms window centered on the acceleration peak. Best-fit line is shown in red. (464 KB PDF) [file pbio.0060215.sg002.pdf]
